# Advanced magnetic resonance imaging (MRI) techniques of the spine and spinal cord in children and adults

**DOI:** 10.1007/s13244-018-0626-1

**Published:** 2018-06-01

**Authors:** M. I. Vargas, B. M. A. Delattre, J. Boto, J. Gariani, A. Dhouib, A. Fitsiori, J. L. Dietemann

**Affiliations:** 1 Division of Neuroradiology, DISIM, Geneva University Hospitals and Faculty of Medicine, Rue Gabrielle-Perret-Gentil 4, 1211, Geneva 14, Switzerland; 2 Division of Radiology, DISIM, Geneva University Hospitals, Geneva, Switzerland; 3 Division of Neuroradiology, Strasbourg University Hospitals, Strasbourg, France

**Keywords:** Spinal cord, Perfusion, Spectroscopy, Magnetic resonance angiography, Diffusion tensor imaging

## Abstract

**Abstract:**

In this article, we illustrate the main advanced magnetic resonance imaging (MRI) techniques used for imaging of the spine and spinal cord in children and adults. This work focuses on daily clinical practice and aims to address the most common questions and needs of radiologists. We will also provide tips to solve common problems with which we were confronted. The main clinical indications for each MR technique, possible pitfalls and the challenges faced in spine imaging because of anatomical and physical constraints will be discussed. The major advanced MRI techniques dealt with in this article are CSF, (cerebrosopinal fluid) flow, diffusion, diffusion tensor imaging (DTI), MRA, dynamic contrast-enhanced T1-weighted perfusion, MR angiography, susceptibility-weighted imaging (SWI), functional imaging (fMRI) and spectroscopy.

**Teaching Points:**

• *DWI is essential to diagnose cord ischaemia in the acute stage*.

• *MRA is useful to guide surgical planning or endovascular embolisation of AVMs*.

• *Three Tesla is superior to 1.5 T for spine MR angiography and spectroscopy*.

• *Advanced sequences should only be used together with conventional morphological sequences*.

## Introduction

Advanced MRI techniques applied to the spinal cord have remained difficult to put into practice until recently. Until now, advanced imaging techniques of the spine have relied on significant contributions from MR physicists to apply them to clinical routine.

These techniques are feasible at 1.5 and 3 T with a clear advantage of 3 T for magnetic resonance angiography (MRA) and spectroscopy.

Characterisation of intramedullary lesions is challenging with conventional sequences, and, on numerous occasions, it is difficult to identify the origin of the lesion, distinguish between inflammation and ischaemia, and correctly date an ischaemic lesion as acute or hyperacute. Conversely, advanced sequences allow a much better depiction of the anatomy, such as diffusion tensor imaging (DTI) for pre-surgical planning of spinal tumours or MRA to accurately localise the shunt and nidus of an AVM.

In this article, we illustrate the main advanced techniques, such as cerebrospinal fluid (CSF) flow, diffusion and diffusion tensor imaging (DTI), dynamic magnetic resonance angiograpy (MRA), dynamic contrast-enhanced T1-weighted perfusion, susceptibility-weighted imaging (SWI), functional imaging and spectroscopy. The main technical challenges faced in spine imaging and the clinical applications of these techniques in children and adults are also discussed.

## Advantages and disadvantages of high field strength

The use of high field strength on MRI in brain imaging has allowed an increased signal-to-noise ratio (SNR) and decreased acquisition time. However, the situation is not the same with advanced sequences in spine and spinal cord imaging. The main challenges faced in spine imaging relate to the small dimensions of the spinal cord (approximately 12 to 14 mm in diameter), inhomogeneity of B0 on T1 imaging, artefacts due to the CSF flow, which are more significant on the dorsal spine, and also artefacts due to breathing, patient motion and swallowing [1]. Magnetic susceptibility artefacts are also important because of interfaces between different structures such as bone, lungs and fat.

Sometimes, high field strength may be a limitation because of the greater effect of susceptibility artefacts (going linearly with the field strength). The distortions of the images are thus important particularly in the spine where the proximity with the lungs creates high susceptibility artefacts. On the other hand, high field strength in the DTI of the cervical spine causes geometric distortion. This has been partially solved by the new sequence types [readout segmentation of long variable echo train (RESOLVE) available on Siemens systems or zoomed EPI available on all systems, for example].

For other sequences such as CSF flow and perfusion imaging, 1.5- or 3-T MR can be used with the same final result. Additionally, high field strength offers a real added value for MRA and spectroscopy; it produces higher SNRs and faster acquisition minimising motion artefacts.

## Advanced sequences

### CSF flow

**Fig. 1:**
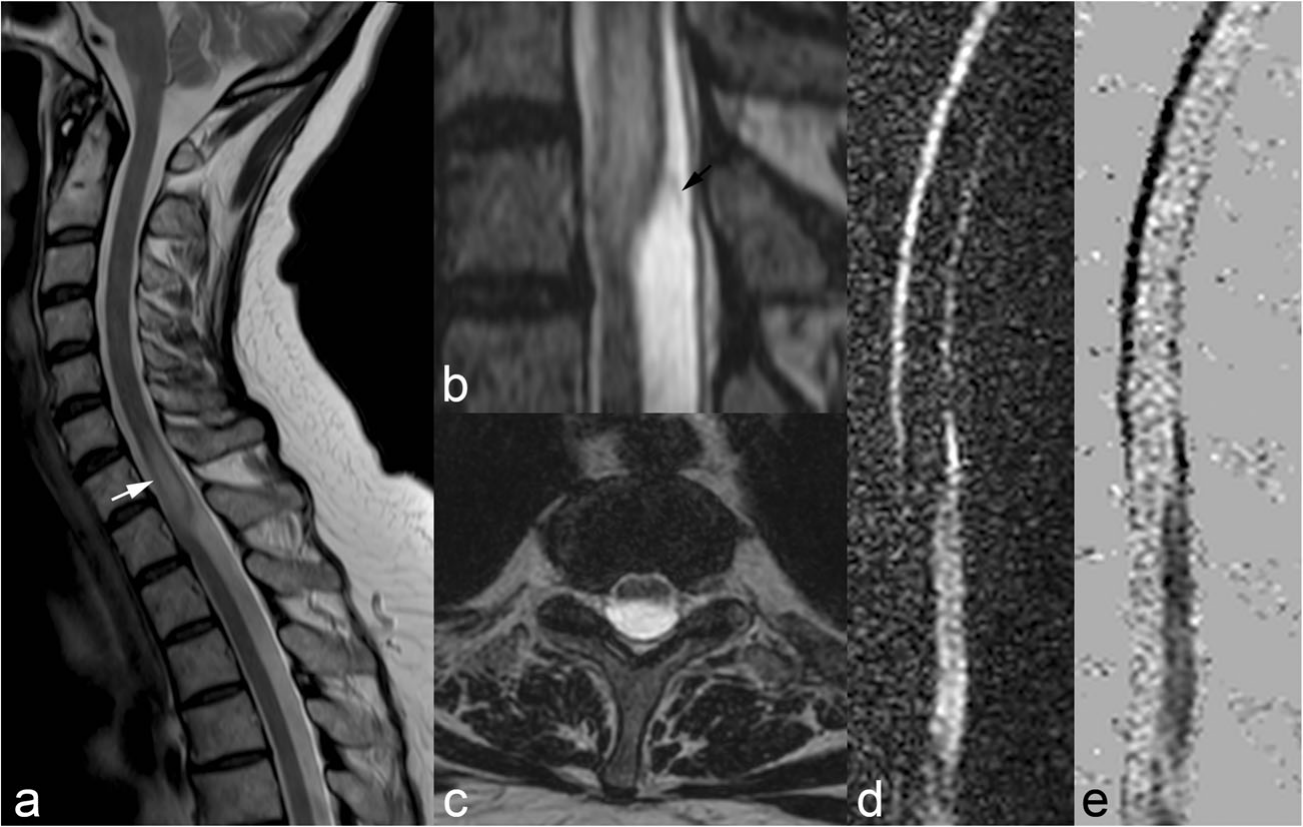
Sagittal (**a**) and axial (**b**) T2WI shows hyperintensity of the cord (arrow), which is deformed (scalpel sign), suggesting an arachnoid web. Sagittal 3D T2WI (**b**) better demonstrates the web (arrow). Sagittal flow sequences (**d**,**e**) show decreased CSF flow posteriorly to the cord due to the arachnoid web

The application of this sequence in spinal cord imaging is for depicting cystic lesions, such as arachnoid or leptomeningeal cysts (Fig. 1), the latter often resulting from haematomas after trauma, which breakdown into haemosiderin and its derivatives and may cause arachnoiditis [2, 3]. CSF flow techniques are usually coupled with T2 high-resolution sequences, which serve the double purpose of helping to better depict lesions and also to generate an anatomical mask.

Phase contrast MRI is a unique approach to measuring flow in vivo. It relies on the principle that motion in a voxel induces additional dephasing of the signal. With an appropriate sequence providing phase images in addition to magnitude images, it is possible to measure the flow velocity in a voxel [4]. A phase encoding gradient can be applied in one of the three directions of space to detect flow in that specific direction. If the flow going through the imaging plane needs to measured, the phase encoding gradient is applied in the slice encoding direction. These phase-contrast sequences require ECG triggering since the blood and CSF flow velocities vary during the cardiac cycle.

Flow measurement is performed in the sagittal plane to visualise in-plane CSF flow. In this case, care has to be taken if the phase encoding direction is chosen since cardiac and respiratory motion are highly deleterious to image quality and flow quantification.

The velocity encoding gradient in the sequence should be set between 10 and 20 cm/s and be increased if aliasing artefacts are observed. Bunck et al. [5] reported 10 cm/s in volunteers and 20 cm/s in patients where a specific pathology could lead to increased velocity due to narrowing of the CSF canal.

Flow can be measured directly in the three spatial directions to improve sensitivity to both the in- and through-plane velocities and also to determine the flow direction. This sequence, which provides data in a 3D volume, is promising but has the disadvantage of long acquisition times (12–14 min versus 3–5 min for the 2D sequence [5]).

### Three-dimensional MR T2 high resolution

**Fig. 2:**
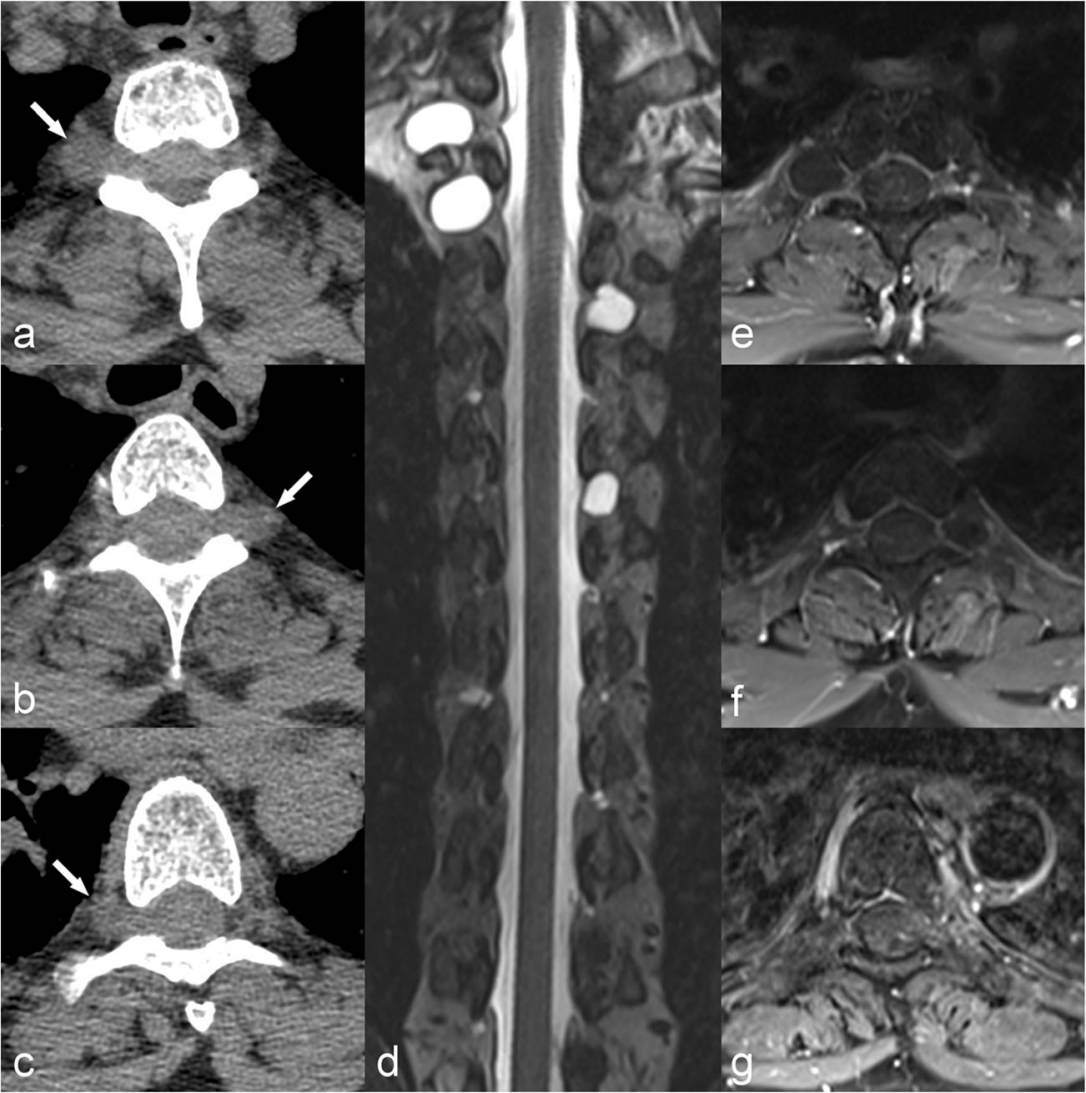
CT scan (**a**,**b**,**c**) performed in a 61-year-old females complaining of back pain after a fall shows several paraspinal lesions (arrows), which are difficult to characterise by this technique. A CISS MR sequence (**d**) clearly shows these to be extradural cysts. Note that there is no enhancement on T1 FS Gd (**e**,**f**,**g**)

Three-dimensional MR T2 high resolution is an isotropic sequence also known as the 3D CISS (constructive interference in steady state), 3D True-FISP (fast imaging with steady-state precession) or FIESTA (fast imaging employing steady-state acquisition) sequence (Fig. 2).

This sequence allows good depiction of smaller structures because of high spatial resolution, high T2 contrast and its isotropic properties, which permit visualisation in three planes without distortion.

The main clinical indications are:

- thin septa in post-traumatic cysts
- walls of arachnoid cysts or arachnoid webs (Figs. 1 and 2)
- inner structure of cystic tumours
- normal and dilated vessels on the surface of the spinal cord in dural fistulas and AVMs
- post-traumatic pseudomeningoceles
- dural breach and CSF leak in hypotension of CSF
- Tarlov cysts

A larger FOV can be obtained in the coronal and sagittal planes, but axial images are also possible.

**Fig. 3:**
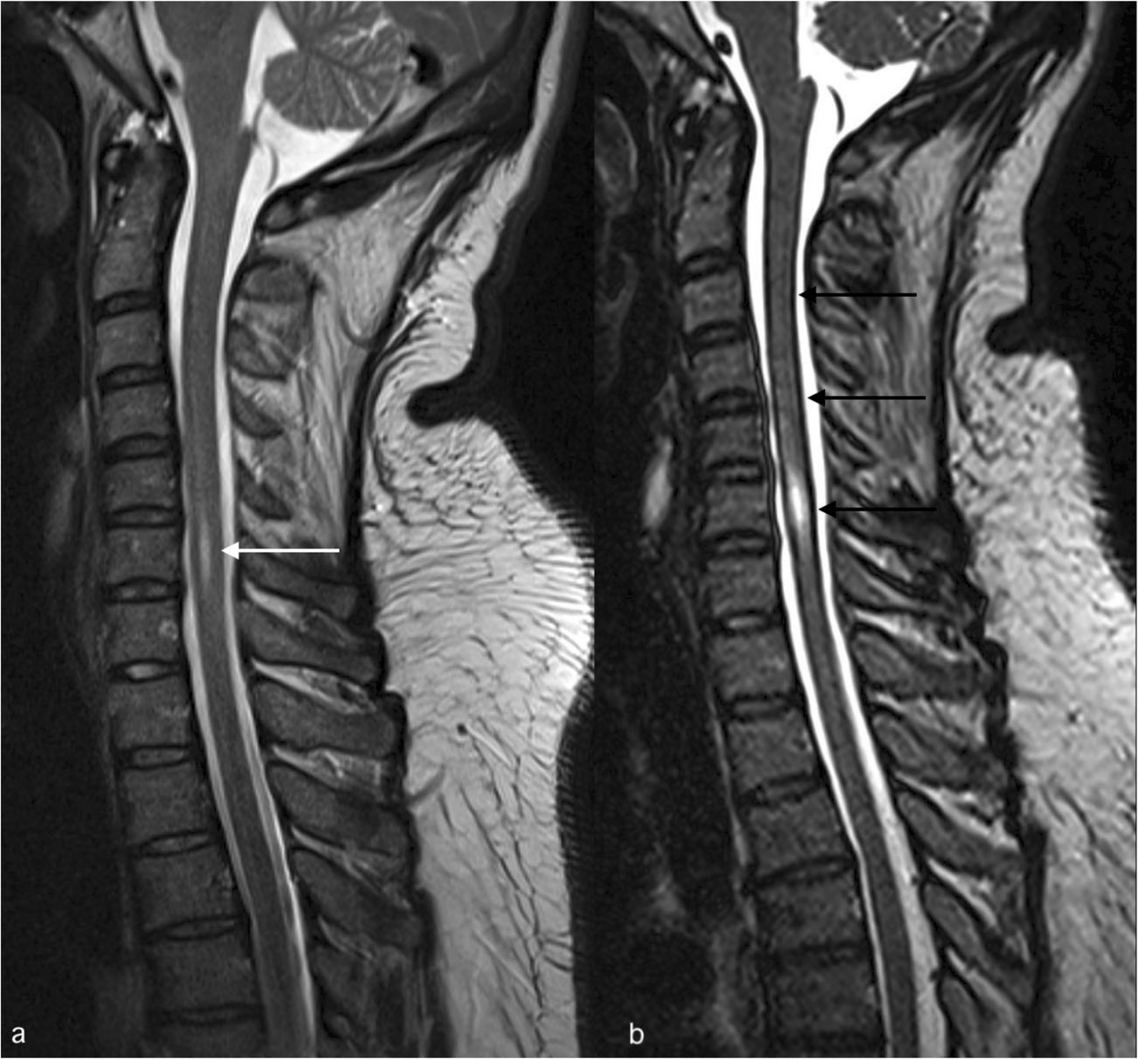
FSE T2 shows an enlarged ependymal canal at the C5 level (white arrow in **a**). Note that the same abnormality appears larger on the SPACE T2 sequence and also the false image of superficial siderosis on the cervical spinal cord (black arrows in **b**)

Other 3D T2 sequences such as space, CUBE or VISTA, which are spin echo sequences, are less sensitive to flow and susceptibility artefacts [6], allowing clear depiction of the nerve root sheaths. The most common pitfall of these sequences is the Gibbs artefact mimicking superficial siderosis [6] (Fig. 3).

### Dynamic MRA

**Fig. 4:**
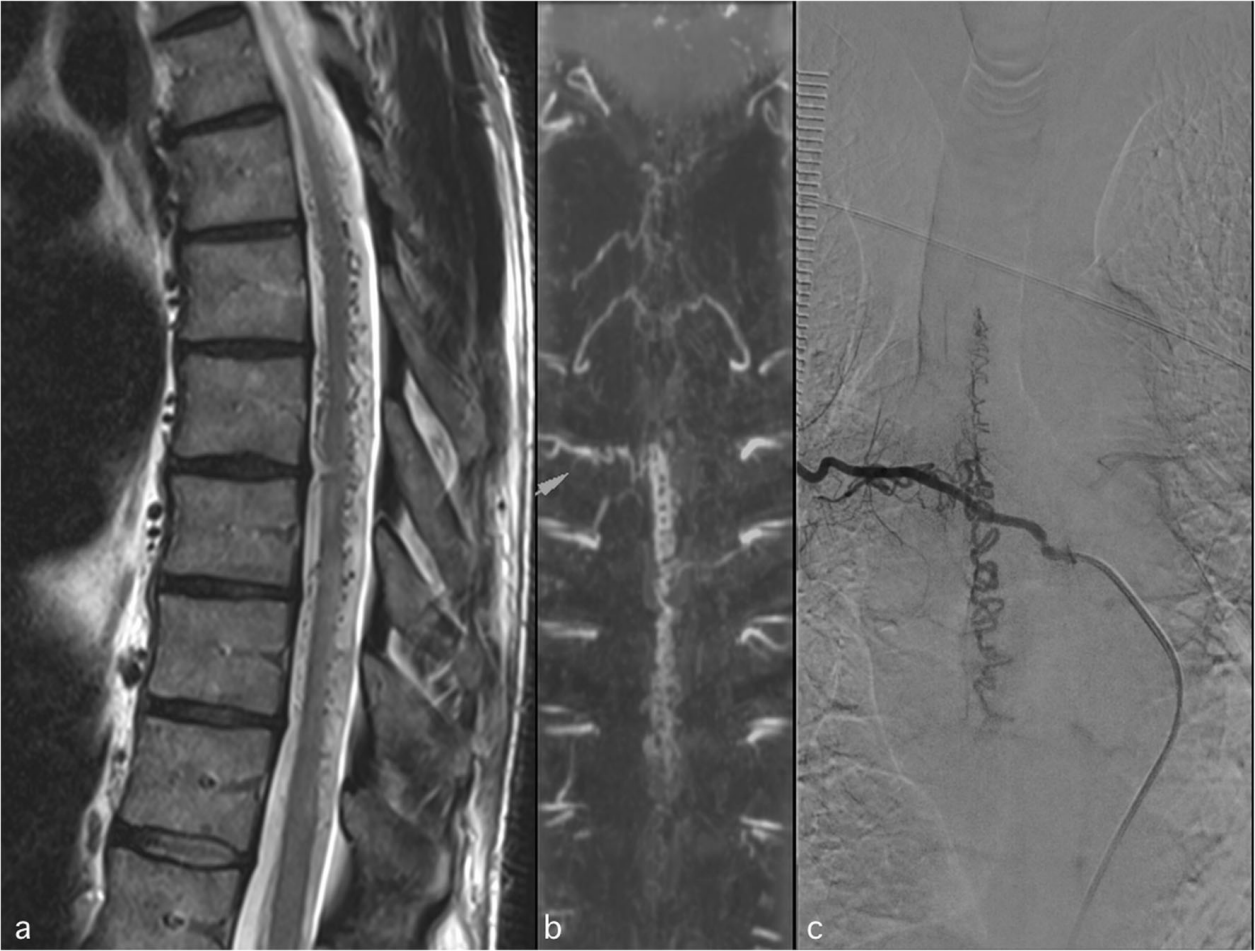
Sagittal T2WI (**a**) demonstrates multiple dilated vessels with corkscrew appearance surrounding the spinal cord (**a**); 3D MRA reformat nicely illustrates the arteriovenous shunt at the level of T6 on the right (arrow in **b**) confirmed by DSA (**c**)

Clinical applications include investigation of vascular malformations such as dural fistulas (Fig. 4) and arteriovenous malformations and identification of the exact location of the artery of Adamkiewicz (AKA) for presurgical planning for tumours to facilitate endovascular treatment. In case of ischaemia, it is very difficult to visualise the thrombus in the vessel lumen, particularly in a small artery such as the AKA. On the other hand, dynamic MRA can be very useful in showing a dissection or a partially thrombosed aneurysm of the aorta. However, it plays no role in the workup of cavernomas as they are angiographically silent. CT is an excellent technique for imaging of large vessels, but it does not allow visualisation of spinal cord ischaemia.

The main challenges of dynamic MRA are the small size of vascular structures in the spine and the dynamic aspect of this sequence. These are also the main reasons why high field strength is useful because of its higher SNR.

There are two techniques for performing dynamic MRA: dynamic sequences with a temporal resolution of approximately 1 min (3 phases: arterial, venous and delayed, and an additional later acquisition at high spatial resolution) [7] and 4D imaging [8].

The first relies on a 3D gradient echo T1-weighted sequence with a field of view comprising the descending aorta as well as the spine in the sagittal plane. The angiography technique uses a noncontrast image that is subsequently subtracted from the arterial phase image. An important aspect is the timing of the imaging. The arterial bolus remains in the arteries for a short period of time; thus, imaging should be done at a precise time to eliminate venous contamination. This timing depends on the injection rate and also on the cardiovascular status of the patient.

The utilisation of blood pool agents or doubly concentrated contrast media may nevertheless facilitate image acquisition and subsequent analysis.

The other technique for MRA relies on a 4D sequence also known as time-resolved angiography with stochastic trajectories (TWIST) [9] or 4D time-resolved MR angiography with a keyhole (4D-TRAK) with a spatial resolution of 1 mm and temporal resolution of approximately 1.3 s.

### Diffusion and diffusion tensor imaging

**Fig. 5:**
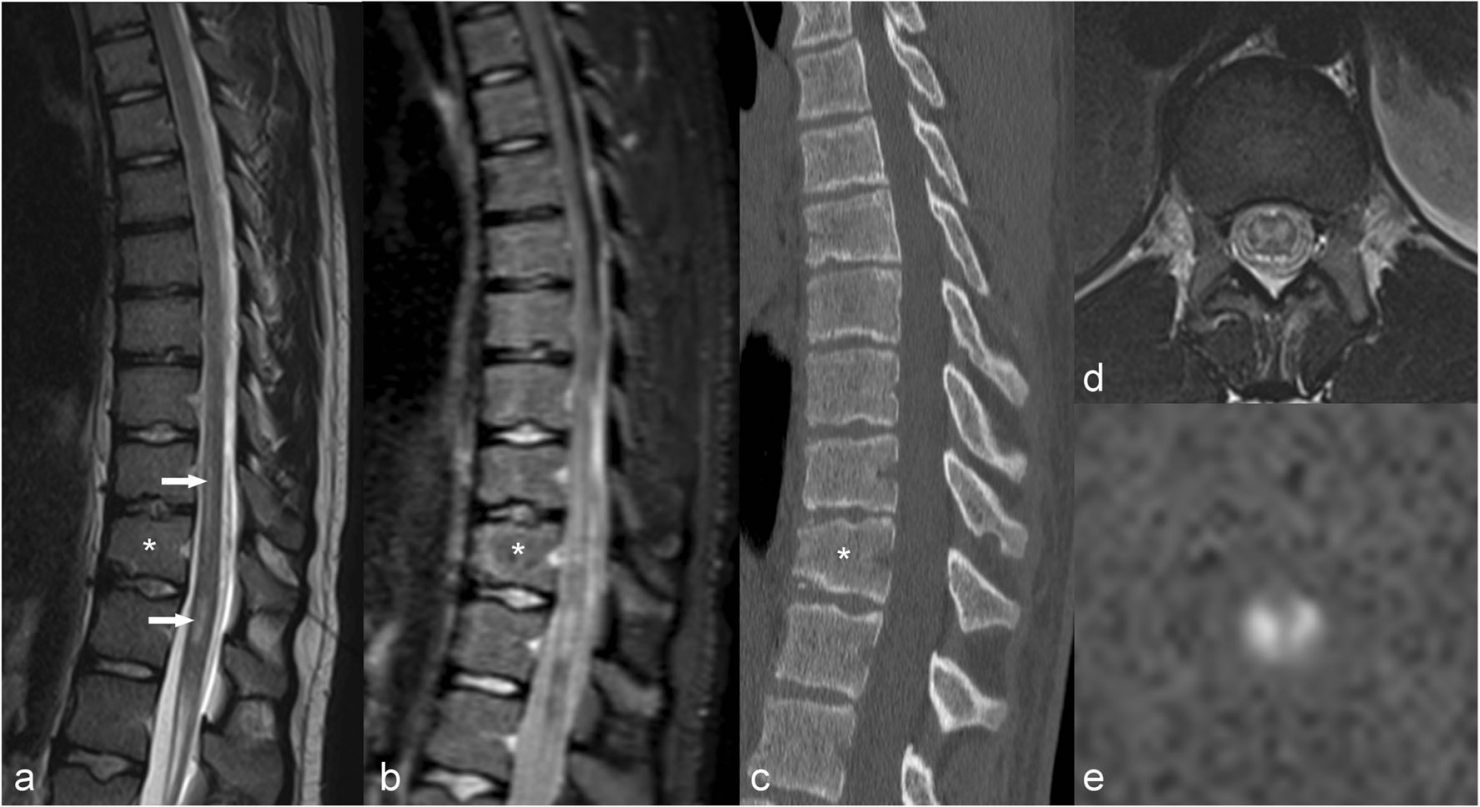
A 14-year-old male who suffered minor trauma. Spine MR performed 24 h later shows increased signal intensity on T2WI (**a**,**d**) (arrows) and DWI (**e**) reflecting ischaemia. Note an acute wedge fracture of T12 (*)

Acute ischaemia is one of the main clinical indications for DWI, is seen as high signal on trace images and decreased ADC without enhancement (Fig. 5), which only appears in the subacute phase [10]. The main causes in adults are atherosclerosis, cardiac surgery and minimally invasive procedures, compression of the radicular artery by a disc [11] and minor trauma to the cervical spine in the setting of degenerative changes.

In children, minor trauma is a cause of ischaemia related to fibrocartilage emboli [12] (Fig. 5) and also arterial spasm. Other causes include traction for scoliosis after orthopaedic surgery [13], complications of cardiac surgery, sickle cell anaemia and umbilical artery catheter in the neonate.

DWI is also used to differentiate between spondylodiscitis and inflammatory degenerative changes [14]. FA and ADC values may be used to predict gain of function in patients with cervical spondylotic myelopathy after decompressive surgery [15].

**Fig. 6:**
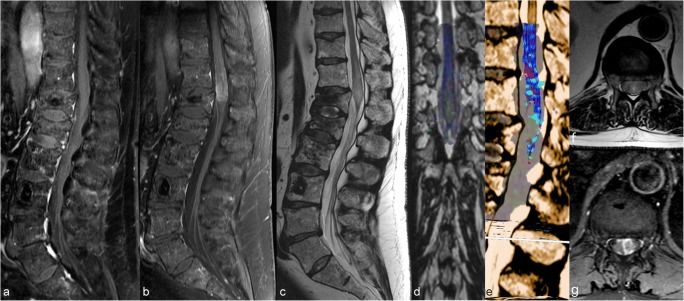
Patient with multiple myeloma and several vertebral fractures, treated by vertebroplasty. No enlargement or enhancement of the conus medullaris is visible (**a**). Spine MR performed approximately 7 months later shows hyperintensity and nodular enhancement of the conus medullaris (**b**,**c**,**f**,**g**). On tractography (**d**,**e**), destruction of the fibers of the conus medullaris can be seen; this translates into a secondary lesion with rapid growth

DTI tractography is used for pre-surgical planning of tumours [16] (Fig. 6) as the generated cartography is the only method allowing the neurosurgeon to visualise the tracts in vivo [17].

Unlike in the brain, diffusion of water molecules in the spinal cord occurs mainly in the cranio-caudal direction [8, 18] because of the lower intracellular water content. This is the main reason why b500 or b900 is used in spinal imaging and not b1000.

DWI and DTI are challenging techniques in spinal imaging for several reasons, including the small size of the cord relative to the brain and respiratory and cardiac motion artefacts. Therefore, spine diffusion imaging requires high spatial resolution, which should be combined with distortion reduction techniques and homogeneous fat saturation. These goals are difficult to achieve with the broadly used single-shot spin echo EPI diffusion sequence, especially when image acquisition is in the sagittal plane, which is preferred for the evaluation of the spine. Specific aspects are (1) fat saturation, (2) imaging distortion and (3) b-values and directions.

1. Conventional fat saturation with spectral selection of the fat peak based on CHESS (chemical shift selective) has the advantage of being fast but often delivers poor results in spine imaging. In dorsal areas, an inversion recovery technique, such as STIR (short tau inversion recovery), is more robust in eliminating the signal from fat and improving image quality. However, this causes a reduction in the signal due to the inversion pulse. A compromise is to use SPAIR (spectral attenuated inversion recovery) preparation, which shows relatively robust saturation provided the shim box is placed correctly in the area of interest avoiding the lungs.

A recently available method to suppress the fat signal is the Dixon technique [19]. It relies on the principle that water and fat do not precess at the exact same frequency and that they can be either in or out of phase after the preselected time to echo. This technique provides very homogeneous fat-saturated images on large fields of view.

(2) Diffusion imaging uses a single-shot EPI sequence. Throughout the long echo train, phase errors will accumulate, resulting in spatial mismatch in the reconstructed image. The longer the echo train and the higher the resolution, the more pronounced the distortions will be. Distortions will also be enhanced because of susceptibility differences between different spinal tissues (bone, intervertebral discs, cerebrospinal fluid, etc.).

Reducing the readout bandwidth minimises this distortion. To achieve this, parallel imaging can be used together with a rectangular field of view. Another option is to choose a transverse orientation with an isotropic voxel resolution. Another alternative to this problem is segmentation of the EPI readout in either the phase or readout direction. A more detailed explanation of distortion reduction in spine diffusion imaging can be found in: [20–22].

(3) b = 500 s/mm^2^ is often chosen at 1.5 T since it produces a sufficient SNR to allow satisfactory interpretation of the images without being too low, and thus too sensitive, to perfusion effects [23]. This value can be increased at 3 T because of the higher SNR.

For DTI, the optimal number of diffusion directions varies depending on the authors [24], a higher number being often preferred. While the minimum number of directions to generate DTI parameters such as FA or MD is six, a more reasonable value would be around 20.

### Dynamic contrast-enhanced T1-weighted perfusion

**Fig. 7:**
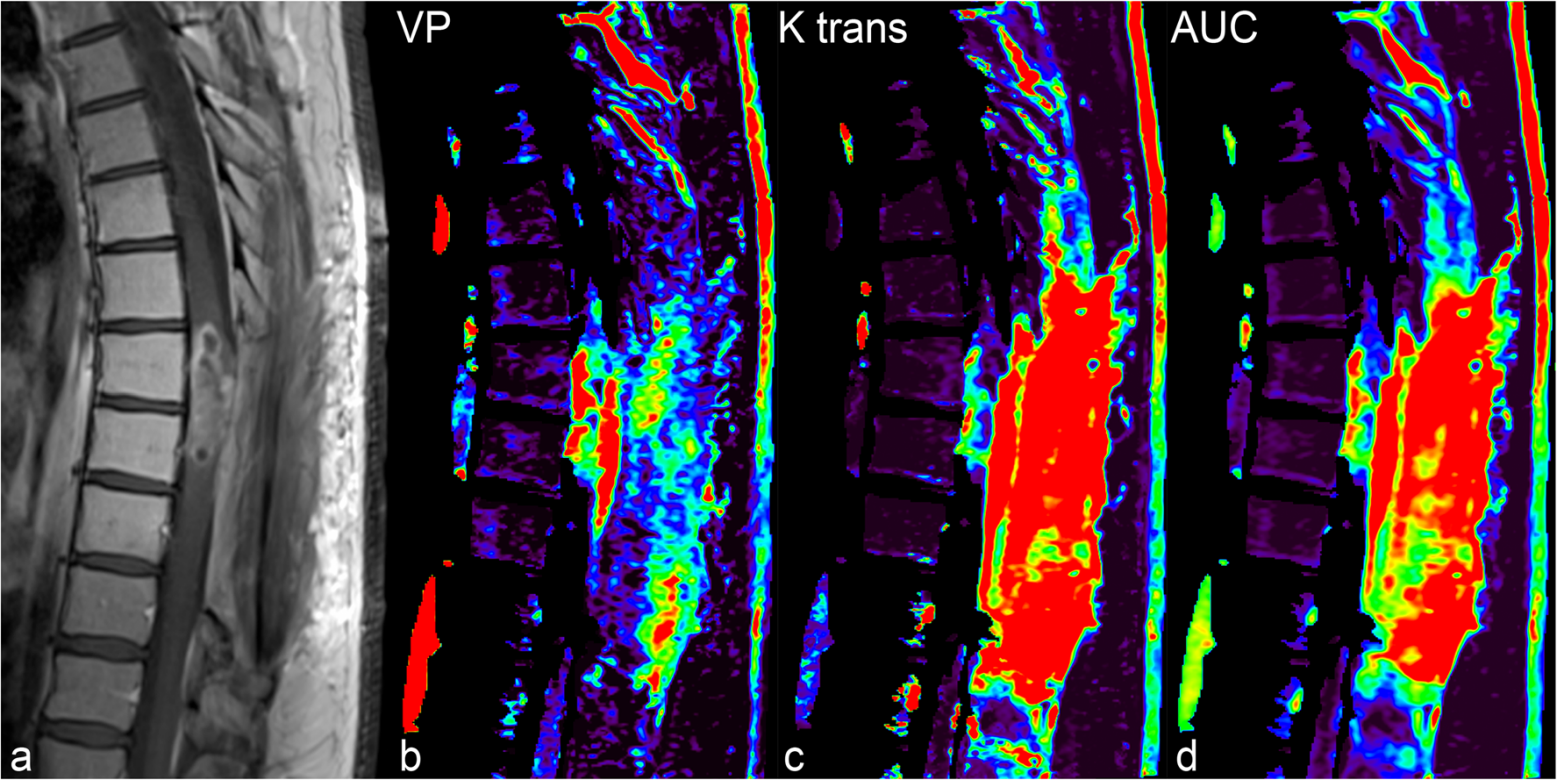
A patient with spinal cord glioblastoma. **a** Sagittal T1 post-gadolinium MR image shows an enhancing intramedullary mass. **b** Corrected Vp (volumetric plasma volume) parameter of T1 perfusion is increased in the mass. **c** and **d** Increased K_trans_ and AUC (area under the curve) in the mass but also in the posterior paraspinal soft tissues reflecting contrast extravasation due to postoperative changes. This case illustrates that T1 perfusion, particularly corrected Vp, allows detection of true tumour hypervascularisation

Dynamic contrast enhancement (DCE) is a technique that allows dynamic visualisation of contrast behaviour in tissues. It is the technique of choice to assess microvascularisation, in particular in the context of tumour growth, because it provides information about the tumour vasculature and the effects of treatment (Fig. 7). This technique is used in brain imaging for tumour characterisation and for distinguishing between radionecrosis and true tumour progression. In spine imaging, DCE is used to characterise tumours and to evaluate extradural spinal metastases and their vascularisation [25], which in turn helps in the selection of patients amenable to endovascular treatment.

The goal of DCE is to quantify tissue permeability with the use of specific models such as the Tofts model or equivalent [26]. This two-compartment model provides physiologically relevant parameters such as the K_trans_ [volume transfer constant between blood plasma and extravascular extracellular space (EES)], K_ep_ (rate constant between EES and blood plasma) and V_e_ (volume of EES per unit volume of tissue, i.e., the volume fraction of the EES).

To generate these parameters, imaging should be performed at relatively high temporal resolution (between 2 to 15 s) and over 5 to 10 min post administration of contrast media. Using a 3D-T1 spoiled gradient recalled echo sequence to dynamically image the contrast arrival and washout is recommended [27]. This sequence is very sensitive to T1 variations and is fast enough to produce a suitable temporal resolution while maintaining a sufficient SNR. For the modelling, it is necessary to convert the signal intensity curve into a Gd concentration curve, which can only be done with knowledge of the T1 values before contrast injection. Usually, the two flip angle method is chosen because it is fast and reliable. This technique has been more widely used in the brain but has also shown promising results in spine imaging, such as preclinical research in spinal cord injury assessment [28, 29].

## Susceptibility weighted imaging (SWI)

SWI is a sequence based on the magnetic susceptibility differences between tissues. Reconstructions of magnitude and phase images are possible. The acquisition of this sequence does not need the administration of contrast medium.

SWI is principally used at the level of the brain to detect micro-haemorrhages, calcifications, iron and deoxy-Hb. Concerning the spine, few articles exist concerning the use of this sequence at 1.5 T. In our opinion, its use is possible but has not been extended to daily clinical practice because of limitations in spatial resolution and multiple artefacts due to phase-encoding directions, bone-tissue interfaces, flow and increased noise. This sequence is particularly sensitive to subtle changes of the local magnetic susceptibility variances, decreased signal-to-noise ratios, etc. [30].

The possibly clinical indications are visualisation of normal venous anatomy [30], identification of haemorrhage, evaluation of the efficiency of treatment of spinal arteriovenous malformations and evaluation of changes in venous oxygenation [31] with SW phase imaging.

### Spectroscopy

Spectroscopy shows the concentration of normal metabolites in a specific anatomic location and changes in those metabolites in case of pathology. Few works have shown the feasibility of spectroscopy in spinal cord imaging. Spectroscopy has, nevertheless, been used to characterise and differentiate tumours [32] from inflammatory pathologies, in amyotrophic lateral sclerosis or in the follow-up of cervical spondylotic myelopathy [33]. Furthermore, Holly et al. [33] showed that spectroscopy may be of value in predicting neurological outcome in patients with cervical spondylotic myelopathy after surgery.

Spectroscopy of the spinal cord presents a real challenge because of the small dimensions of the cord, flow artefacts, motion artefacts during the cardiac and respiratory cycles, the deep anatomical location [34] and B0 inhomogeneity, which is particularly deleterious to spectroscopy of the spine. For this reason, B0 shimming is essential. Saturation bands and pulse triggering [32] are used to reduce CSF flow artefacts.

The sequence used is PRESS (point-resolved spectroscopy), which produces better results with a long TE of around 135 or 280 ms than with short TE below 40 ms. Currently, this technique is used exclusively in research.

## Functional imaging (fMRI)

fMRI provides information concerning spinal cord motor function. The sequence used is essentially the same as that on the brain; however, modifications are necessary because of the size of the spinal cord. Spinal fMRI illustrates neuronal function indirectly by changes in blood flow and blood oxygen levels (potential clinical indication of fMRI) [35].

This tool is only used for research purposes.

The potential clinical applications are: to determine preserved motor function in patients with spinal injury, to plan treatment or to evaluate the treatment response of tumours to understand the physiopathology of cervical headaches and to monitor functional changes in diseases such as multiple sclerosis and amyotrophic lateral sclerosis [35].
